# Measuring human capital: a systematic analysis of 195 countries and territories, 1990–2016

**DOI:** 10.1016/S0140-6736(18)31941-X

**Published:** 2018-09-24

**Authors:** Stephen S Lim, Rachel L Updike, Alexander S Kaldjian, Ryan M Barber, Krycia Cowling, Hunter York, Joseph Friedman, R Xu, Joanna L Whisnant, Heather J Taylor, Andrew T Leever, Yesenia Roman, Miranda F Bryant, Joseph Dieleman, Emmanuela Gakidou, Christopher J L Murray

**Affiliations:** **Institute for Health Metrics and Evaluation, University of Washington, Seattle, WA, USA** (Prof S S Lim PhD, R L Updike BA, A S Kaldjian MSc, R M Barber BS, K Cowling PhD, H York BA J Friedman MPH, R Xu BS J L Whisnant MPH, H J Taylor BA, A T Leever BS, Y Roman MLIS M F Bryant MPH, J Dieleman PhD, Prof E Gakidou PhD Prof C J L Murray DPhil); **and David Geffen School of Medicine at University of California Los Angeles Los Angeles, CA, USA** (J Friedman)

## Abstract

**Background:**

Human capital is recognised as the level of education and health in a population and is considered an important determinant of economic growth. The World Bank has called for measurement and annual reporting of human capital to track and motivate investments in health and education and enhance productivity. We aim to provide a new comprehensive measure of human capital across countries globally.

**Methods:**

We generated a period measure of expected human capital, defined for each birth cohort as the expected years lived from age 20 to 64 years and adjusted for educational attainment, learning or education quality, and functional health status using rates specific to each time period, age, and sex for 195 countries from 1990 to 2016. We estimated educational attainment using 2522 censuses and household surveys; we based learning estimates on 1894 tests among school-aged children; and we based functional health status on the prevalence of seven health conditions, which were taken from the Global Burden of Diseases, Injuries, and Risk Factors Study 2016 (GBD 2016). Mortality rates specific to location, age, and sex were also taken from GBD 2016.

**Findings:**

In 2016, Finland had the highest level of expected human capital of 28·4 health, education, and learning-adjusted expected years lived between age 20 and 64 years (95% uncertainty interval 27·5–29·2); Niger had the lowest expected human capital of less than 1·6 years (0·98–2·6). In 2016, 44 countries had already achieved more than 20 years of expected human capital; 68 countries had expected human capital of less than 10 years. Of 195 countries, the ten most populous countries in 2016 for expected human capital were ranked: China at 44, India at 158, USA at 27, Indonesia at 131, Brazil at 71, Pakistan at 164, Nigeria at 171, Bangladesh at 161, Russia at 49, and Mexico at 104. Assessment of change in expected human capital from 1990 to 2016 shows marked variation from less than 2 years of progress in 18 countries to more than 5 years of progress in 35 countries. Larger improvements in expected human capital appear to be associated with faster economic growth. The top quartile of countries in terms of absolute change in human capital from 1990 to 2016 had a median annualised growth in gross domestic product of 2·60% (IQR 1·85–3·69) compared with 1·45% (0·18–2·19) for countries in the bottom quartile.

**Interpretation:**

Countries vary widely in the rate of human capital formation. Monitoring the production of human capital can facilitate a mechanism to hold governments and donors accountable for investments in health and education.

**Funding:**

Institute for Health Metrics and Evaluation.

## Introduction

Human capital refers to the attributes of a population that, along with physical capital such as buildings, equipment, and other tangible assets, contribute to economic productivity.^1^ Human capital is characterised as the aggregate levels of education, training, skills, and health in a population,^2^ affecting the rate at which technologies can be developed, adopted, and employed to increase productivity.^3^ The World Bank has brought new attention to this topic through its recently introduced Human Capital Project,^4^ which aims to “understand the link between investing in people and economic growth, and to accelerate financing for human capital investments.” A basic input needed for this aim to be fulfilled is an internationally comparable index of human capital, which currently does not exist. This study seeks to fill this global measurement gap.^3^

Although evidence supports human capital as a driver of growth, the World Bank has argued that investments in human capital are too low in low-income and middle-income countries.^5^ Much of the World Bank’s investments focus on physical rather than human capital.^5^ Only 1·5% of the World Bank International Development Association concessional grants are for health and 1·9% are for education.^6^ As countries graduate to borrowing from the non-concessional International Bank for Reconstruction and Development framework, the shares for health increase to 4·2% and to 5·2% for education.^6^ A focus on building physical assets might also be driven by time horizons; such projects can yield returns sooner than investing in children’s health and education, and the political process in many nations might reward short-run returns.^6^

Research in context

**Evidence before this study**

Previous studies have examined the association between a range of dimensions of human capital and economic growth. These studies have shown that the average number of years of completed schooling is associated with subsequent economic growth and that incorporation of measures of the distribution of education might explain more of this variation. More recent analyses from the past 5–10 years that use performance on international student assessments as a measure of educational quality or learning find it to be a more predictive measure of economic growth than attainment alone. Far fewer efforts have been made to expand the measurement of human capital so that it also encompasses health; however, these studies suggest that an expanded measurement might also be important for understanding economic growth. Despite the accumulated evidence of the associations between the core dimensions of human capital—education and health—and economic growth, no comprehensive measure presently exists for all countries globally.

**Added value of this study**

This study provides a new measure of expected human capital for 195 countries, consisting of four components: educational attainment, learning, health, and survival, based on a systematic analysis of all available data. This measure, in units of health, education, and learning-adjusted expected years lived between age 20 and 64 years, is estimated each year from 1990 to 2016 and can be updated annually. Compared with existing metrics of human capital, this more comprehensive measure provides a detailed characterisation of these differences across countries and over time, revealing marked variations in expected human capital for children born in different countries and differential progress in the improvement of expected human capital over the past 25 years. An inconsistent gender differential exists—for countries below approximately 10 years of expected human capital, this tends to be higher in males; for countries above this level, it is higher in females.

**Implications of all the available evidence**

Human capital is an important factor in economic development that requires improved metrics and regular monitoring. The systematic analysis of data on four components— educational attainment, learning, health, and survival— establishes the feasibility of an annual measurement of expected human capital, providing a means to monitor and assess investments in health and education. This more comprehensive measure of human capital has revealed variability across countries in building human capital that is independent of baseline levels of health and education, suggesting that building human capital is amenable to policy intervention.

Despite the inclusive scope of the theory of human capital, much of the initial research has focused on the average number of years of completed schooling,^7^ found to be associated with subsequent economic growth,^8,9^ although the association is not consistent.^3^ Research that uses the distribution of education has found that it might explain more variation in economic growth than a simple average.^10^ In the past 5–10 years, analyses of around 50 countries^11–13^ that further take into account the quality of education or learning, with the use of performance on international student assessments, find this measure is even more predictive of economic growth. Efforts to expand the measurement of human capital to also encompass functional health have been far fewer^14,15^ but suggest that health could also be important for under- standing economic growth.^3^

Underinvestment in people might also be driven by a paucity of data; presently, regular and comparable reports on the rates of formation of human capital across all countries do not exist.^5^ Monitoring the expected formation of human capital in the next generation, as a measure of the effect of near-term investments in health and education, could facilitate a mechanism to hold countries and donors accountable to their populations for these investments.^5^

Building on past efforts, we have produced a measure of human capital that incorporates educational attainment, education quality or learning, functional health, and survival for 195 countries, by age and sex, from 1990 to 2016. For each country, we estimated the expected years of human capital, defined for each birth cohort as the expected years lived from 20 to 64 years of age and adjusted for educational attainment, learning, and functional health, if exposed to period-specific, age- specific, and sex-specific rates of mortality, educational attainment, learning, and functional health status.

## Methods

### Overview

We did a systematic analysis of available data for 195 countries from 1990 to 2016 to measure educational attainment, by sex and 5-year age groups (from 5 to 64 years) for the in-school and working-age population, and learning, as measured by performance on stand-ardised tests of mathematics, reading, and science by 5-year age groups (from 5 to 19 years) for school-aged children. We constructed a measure of functional health status using the prevalence, by 5-year age groups, of seven health conditions for which evidence suggests a link to economic productivity using estimates from the Global Burden of Diseases, Injuries, and Risk Factors Study (GBD) 2016.^16^ We also used mortality rates specific to location, age, sex, and year from GBD 2016.^17^

Using these four dimensions—educational attainment, learning, functional health, and survival—we constructed an indicator of expected human capital that is sensitive to recent investments in health and education. Expected human capital is defined as the expected years lived from age 20 to 64 years and adjusted for educational attainment, learning, and functional health, measured in units of health, education, and learning-adjusted expected years lived between age 20 and 64 years. Expected human capital is calculated by exposing a hypothetical birth cohort to educational attainment, learning, functional health status, and mortality rates specific to time period, age, and sex. The measure is analogous to health-adjusted life expectancy. Expected human capital was calculated as follows:

$$\left(\frac{\sum_{x=20}^{64}\;nL_{xt}FH_{xt}}{l_{0}}\right)\left(\frac{\sum_{x=5}^{24}\;Edu_{xt}Learn_{xt}}{18}\right)$$

where *nL_xt_* is the expected years lived in an age group x, for year t, in which age groups are defined as birth–6 days, 7–27 days, 28 days–1 year, 1–4 years, and 5-year age groups thereafter; *FH_xt_* is the functional health status in an age group *x*, in year t, transformed to a 0–1 scale; l0 is the starting birth cohort*; Edu_xt_* is the years of education attained during an age group *x*, for year t; and *Learn_xt_* is the average standardised test score in an age group *x*, for year t, transformed to a 0–1 scale.

In other words, for a birth cohort born, for example, in the year 2000, we exposed the birth cohort to age and sex-specific mortality rates for the year 2000 from birth to 64 years. For each 5-year group from 20 to 64 years, we adjusted years lived by the cohort in each interval for age-specific and sex-specific functional health status and calculated the number of adjusted years lived from 20 to 64 years. From 5 to 24 years, we computed the expected number of learning-adjusted years of education by exposing the cohort to age-specific and sex-specific educational attainment rates adjusted for learning estimated for the year 2000. We summed and divided these estimates by the maximum possible learning adjusted years of education; we used 18 years, which is the commonly used maximum for educational attainment data.^18^ We used the subsequent ratio to adjust the health adjusted years lived from 20 to 64 years to produce the measure of expected human capital.

We did a sensitivity analysis (appendix) in which we took the mean instead of the product of learning-adjusted educational attainment and functional health when computing expected human capital.

### Educational attainment

Estimates of average years of education were based on a compilation of 2522 censuses and household surveys. These data and the methods hereafter build on an approach used to produce a previously published dataset of international educational attainment.^19^ All data were top-coded to 18 years of education based on the practices of a common data provider.^18^ Each data source included information on the distribution of educational attainment by country, year, sex, and 5-year or 10-year age group. When years of schooling data were available only for multiyear bins—eg, the fraction of the population with between 6 and 9 years of completed education—we used a database of 1792 sources reporting single years of completed schooling to split these binned data into single-year distributions from 0 to 18 years on the basis of the average of the 12 closest distributions in terms of geographical proximity and year. From each of the subsequent data sources, we calculated the mean years of schooling by age and sex.

In the next step, we used age-cohort imputation to project observed cohorts through time, exploiting the relative constancy of education levels after 25 years of age. For any datapoint representing a cohort aged 25 years or older, we extrapolated the data forward and backward so that it was represented in all year-age combinations for that cohort. For example, a datapoint reflecting a cohort aged 35–39 years in 2000 was projected forward for people aged 40–44 years in 2005, aged 45–49 years in 2010, and so on. It was also projected backward for people aged 30–34 years in 1995 and people aged 25–29 years in 1990. After imputation, we fitted age–period models on all original input data and the imputed cohort data to estimate a complete single-year series of educational attainment from 1950 through to 2016 by age, sex, and location. We separately calculated for each sex and GBD region the mean level of educational attainment of the country, age, sex, and year-specific population (*Edu*,_c,a,s,t_), which was estimated as:

$$logit\;\left(\frac{Edu_{c,a,s,t}}{Edu_{\max_{a}}}\right)=\beta_{{}_{s,r}}\;Year\;+\;\delta_{{}_{s,r}}\;Age\;+\;I_{{}_{s,r}}\;+\;\alpha_{{}_{c,s}}$$

where *Edu_maxa_* is the maximum mean educational attainment for each age group, defined as three for ages 5–9 years, eight for ages 10–14 years, 13 for ages 15–19 years, and 18 for all age groups 20–24 years and older; βs,r is a sex-specific and region-specific intercept; δ_s,r_ captures the linear secular trend for each sex and region; I_s,r_ is a natural spline on age to capture the non-linear age pattern by sex and region, with knots at 15 and 25 years; and α_c,s_ is a country-sex-specific random intercept.

Finally, we used Gaussian process regression (GPR) to smooth the residuals from the age–period model, accounting for uncertainty in each datapoint. GPR also synthesises both data and model uncertainty to estimate uncertainty intervals.

### Learning

Our estimates of learning or education quality are based on a systematic analysis of student testing data from major international assessments and national continuing assessments of education progress. These data comprise1894 tests, covering 4345 location–subjects (mathematics, science, and reading) across 295 unique locations (132 countries and 163 subnational locations).

Our testing database contains a comprehensive record of learning scores for school-aged children aged 5–19 years. Four major programmes provide extensive data: the Programme for International Student Assessment, which began in 2000 and now tests students in 73 countries on a 3-year cycle;^20^ the Progress in International Reading Literacy Study (PIRLS), which covered 50 countries in the 2016 iteration;^21^ the Trends in International Mathematics and Science Study (TIMSS), of which the latest round in 2015 covered 57 countries;^22^ and several tests from the International Association for the Evaluation of Educational Achievement.^23,24,25^ In addition to these programmes, we also used regional testing programmes, including the Southern and Eastern Africa Consortium for Monitoring Educational Quality,^26^ the Latin American Laboratory for Assessment of the Quality of Education,^27^ and the Programme d’ Analyse des Systèmes Educatifs de la Confem;^28^ national standardised testing programmes, such as the US National Assessment of Education Progress,^29^ and the India National Achievement Survey;^30^ and representative studies measuring intelligence quotient (IQ) in school-aged children that largely included the Wechsler Intelligence Scale for Children,^31^ the Raven’s Standard Progressive Matrices,^32^ and the Peabody Picture Vocabulary test.^33^ This database provides the most extensive geographical distribution and compilation of long-term temporal trends to date. Unlike several other studies,^34–38^ which used similar data, we kept scores in different school subjects (ie, mathematics, reading, and science) separate. We also maintained data on the year the tests were done to understand trends through time and included demographic information such as grade level (for implied age) and sex.

To generate comparable measures from these different tests, we rescaled subject-specific test scores to a common reference test scale using linear regression, building on previous approaches.^39^ We used TIMSS mathematics and science tests and PIRLS reading tests as the reference scale because they are large, international tests that cover most geographical regions and all three major testing subjects, and are already standardised to each other.^40^ We implemented the rescale using all available data matched by country and approximate year for the reference tests and alternative tests.

To estimate test scores for all countries, years, and ages (5-year age groups from 5 to 19 years), we used spatiotemporal Gaussian process regression^41^ using per capita mean years of education as a predictor (β=5·7, p=0 for boys; β=5·8, p=0 for girls), and with maths, science and reading test scores given equal weight to generate a combined learning measure ranging from 0 to 1000. This method draws strength across space, time, and age, incorporates both data and model uncertainty, and produces a full-time series of estimates for all geographies with the use of covariate relationships and spatial and temporal patterns in residuals.

Finally, we rescaled this measure to a 0–1 scale, with 1 set to one SD above the mean score (a score of 600) on the original TIMSS exam,^40^ approximately the highest estimated average test score in any country.

### Functional health status

For functional health status relevant to economic productivity, we used the prevalence of seven diseases and impairments identified in policy trials or observational studies to be related to learning or productivity (appendix). These include wasting, measured as the proportion of the population younger than 5 years below two SDs of the reference mean weight for height;^42^ stunting, measured as the proportion of the population younger than 5 years below two SDs of the reference height for age;^42^ anaemia, measured as the proportion of each age–sex group with a haemoglobin concentration defined by WHO as mild, moderate, or severe anaemia;^43^ cognitive impairment, measured as the proportion of the population with moderate, severe, or profound developmental delay;^44^ vision loss, defined as the proportion of the population with moderate or severe vision impairment or blindness;^45^ hearing loss, defined by WHO as the proportion of the population with hearing loss greater than 40 dB in the better-hearing ear (30 dB in children);^46^ and infectious disease prevalence, with the use of three infectious disease aggregations from GBD 2016 classification, which includes HIV/AIDS, tuberculosis, malaria, neglected tropical diseases, diarrhoea, and several other common infectious diseases.^47^

We combined these seven functional health status outcomes into a single measure using principal components analysis (PCA). In the first step, we used country-specific prevalence rates of anaemia, vision loss, hearing loss, intellectual disability, and years lived with disability per capita from infectious disease for 5-year age groups (20–64 years), from 1990 to 2016. Because stunting and wasting are measured only in children younger than 5 years, we used the time-period measure of prevalence in children for these two conditions. We rescaled each of the seven conditions such that 0 represented the first percentile and 1 represented the 99th percentile observed across all age, sex, and country groups. We then applied PCA on the age-standardised value of the rescaled health conditions for the ages 20–64 years. Following standard practice, we selected the first n components of the PCA such that the sum of the variance explained by the components was greater than 80%.^48^ In this case, the first component explained more than 85% of the variance. We determined weights for each condition by taking the average loading across factors, weighted by the explained variance. We rescaled this vector of weights so that it was equal to one. We then calculated the health component score for each observation specific to age, sex, country, and year by applying these PCA-generated weights to the seven component prevalence values.

### Survival

We estimated expected years lived between ages 20 and 64 years using sex-specific and age-specific mortality rates by country and year, produced for GBD 2016. This estimation procedure used a wide range of sources including, but not limited to, adjusted data from vital and sample registration systems and birth histories and sibling survival data collected in household surveys to populate abridged life tables and compute expected years lived by 5-year age groups. These methods are described in detail in a previous publication.^17^

### Uncertainty analysis

We estimated uncertainty in the measure of expected human capital by computing 1000 estimates of expected human capital using 1000 draws from the posterior distribution of each of the four components (educational attainment, learning, functional health status, and survival). The posterior distribution of each of the four components reflects both the variance of the input data and predictors used in the estimation model of each component.

### Associations between expected human capital and gross domestic product

We examined the association between GDP per capita and expected human capital in two ways, using GDP per capita data from a recently published health financing dataset.^49^ First, we plotted the cross-sectional association between GDP per capita and expected human capital, by country, in 1990 and 2016, using GDP per capita in both log and level space. Second, for countries in each quartile of expected human capital in 1990 and 2016, we computed the median and IQR of GDP per capita in 1990 and 2016. For quartiles formed by the absolute change in expected human capital between 1990 and 2016, we also computed the median and IQR of the annualised rate of change in GDP per capita from 1990 to 2016.

## Results

### Levels and trends in expected human capital

**Figure 1:**
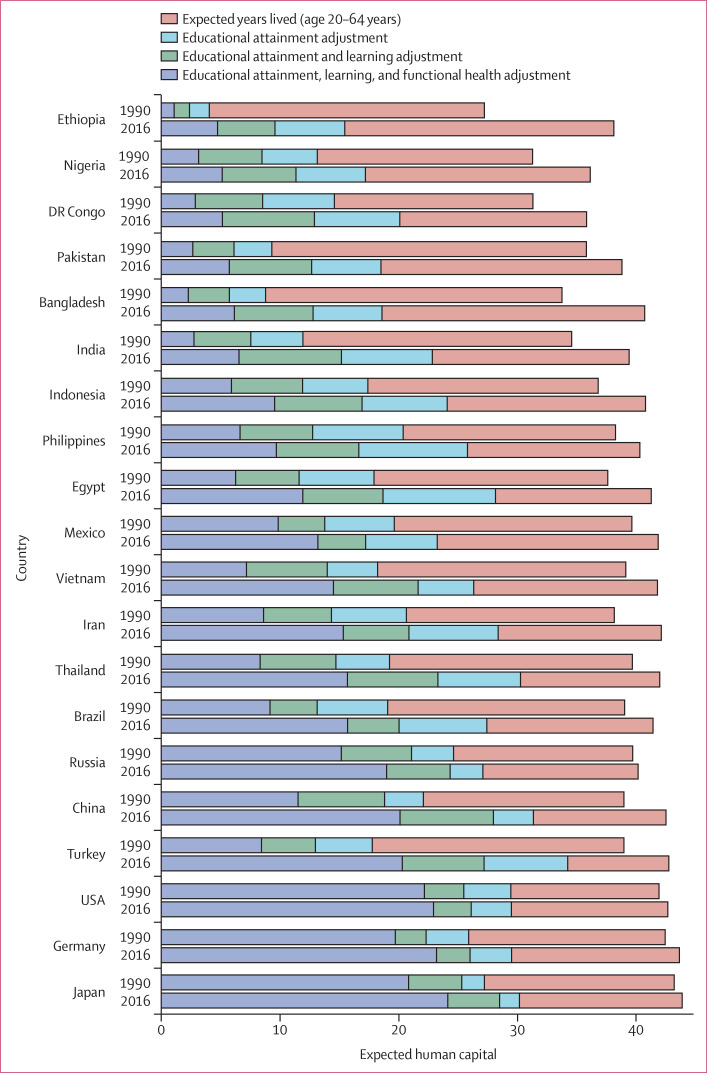
Expected human capital in 1990 and 2016 for the 20 largest countries in the world, by 2016 total population The effect of progressively combining the four components of human capital—expected years lived from 20 to 64 years of age, mean years of education, learning, and functional health status—in the largest 20 countries in the world differs by country and year.

After the effect of taking all four components of expected human capital into consideration for the 20 largest populations in the world (figure 1), Japan in 2016 had the highest expected human capital of 24·1 expected years lived (95% UI 23·2–25·0) from 20 to 64 years of age, adjusted for educational attainment, learning, and functional health. This value comes from Japan having 43·9 expected years lived (95% UI 43·8–43·9) from 20 to 64 years based on age-specific mortality rates in 2016, expected educational attainment of 12·4 years (12·0–12·8) out of a maximum possible of 18 years, a learning score of 0·95 (0·93–0·96), and a functional health score of 0·85 (0·84–0·85). On the low end in 2016 was Ethiopia, where expected human capital, despite substantial progress, was still less than 5 years: expected years lived from 20 to 64 years was 38·1 years (95% UI 37·4–38·9), educational attainment was only 7·3 years (6·0–8·6), the learning score was only 0·62 (0·61–0·63), and the functional health score was 0·49 (0·46–0·52). Differences in the change in expected human capital between 1990 and 2016 highlight large variations in progress in producing human capital across these countries. Showing the most dramatic increase, Turkey in 2016 had expected years lived from 20 to 64 years of 42·7 years (95% UI 42·4–43·1), expected years of schooling of 14·4 years (13·4–15·2), a learning score of 0·79 (0·78–0·80), and a functional health score of 0·75 (0·71–0·77), yielding expected human capital of 20·3 years (18·6–21·8) in 2016, up from 8·4 years (7·9–8·9) in 1990.

**Figure 2:**
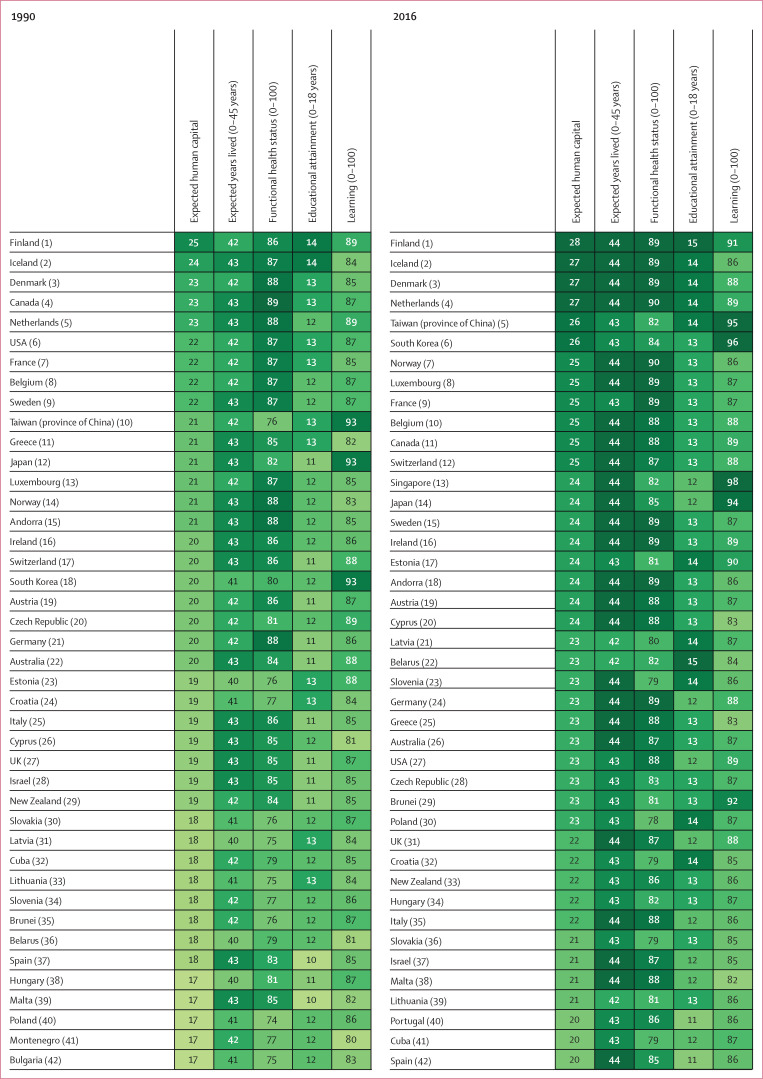

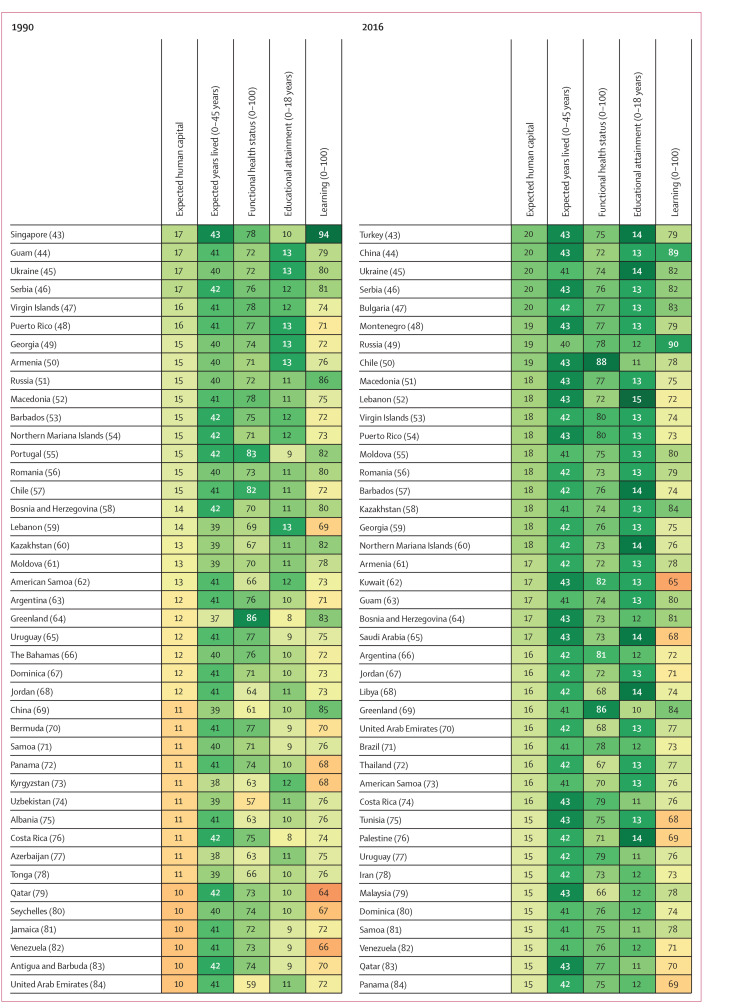

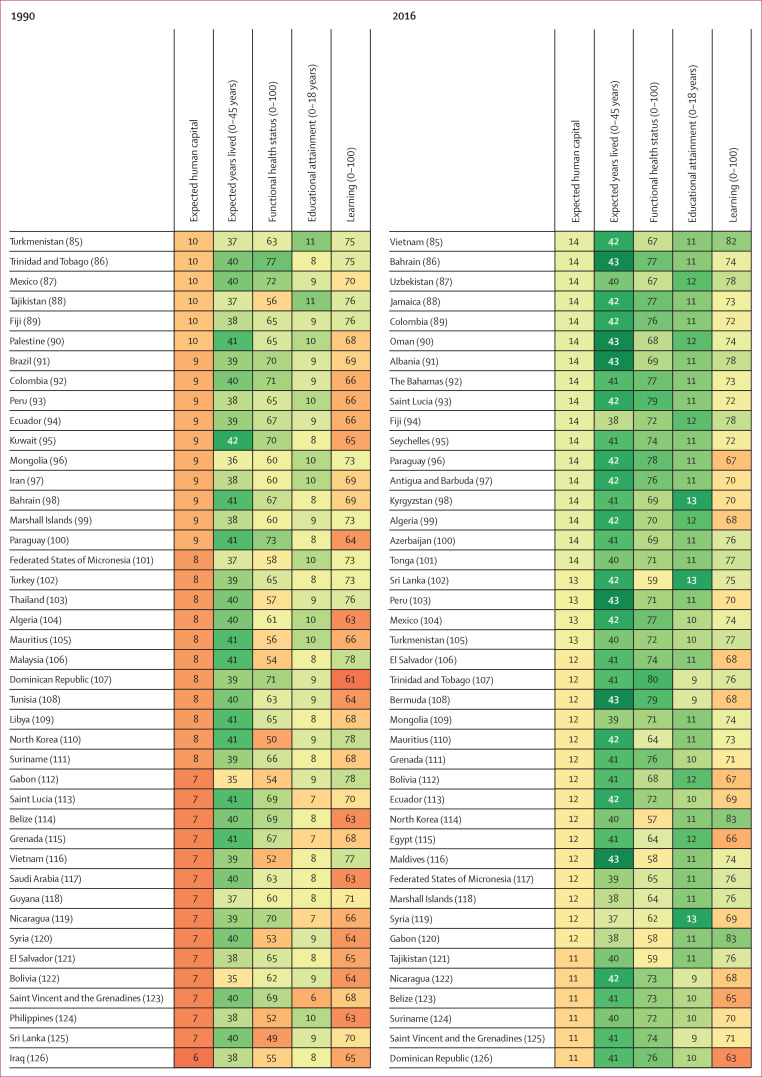

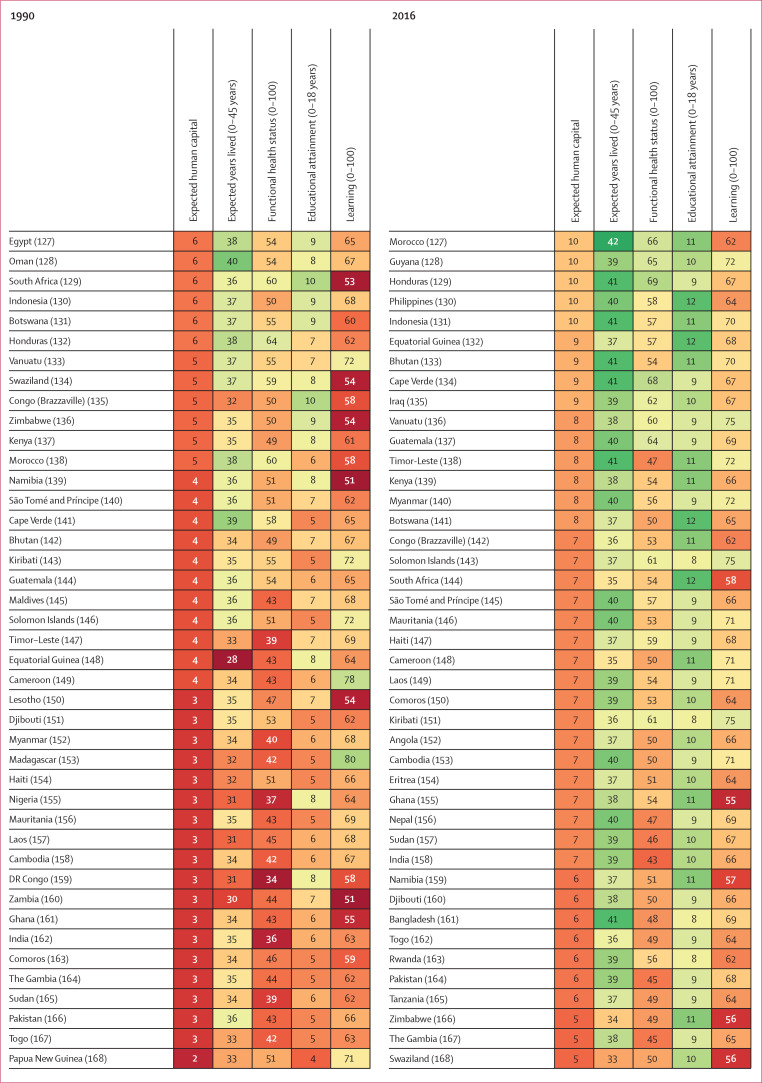

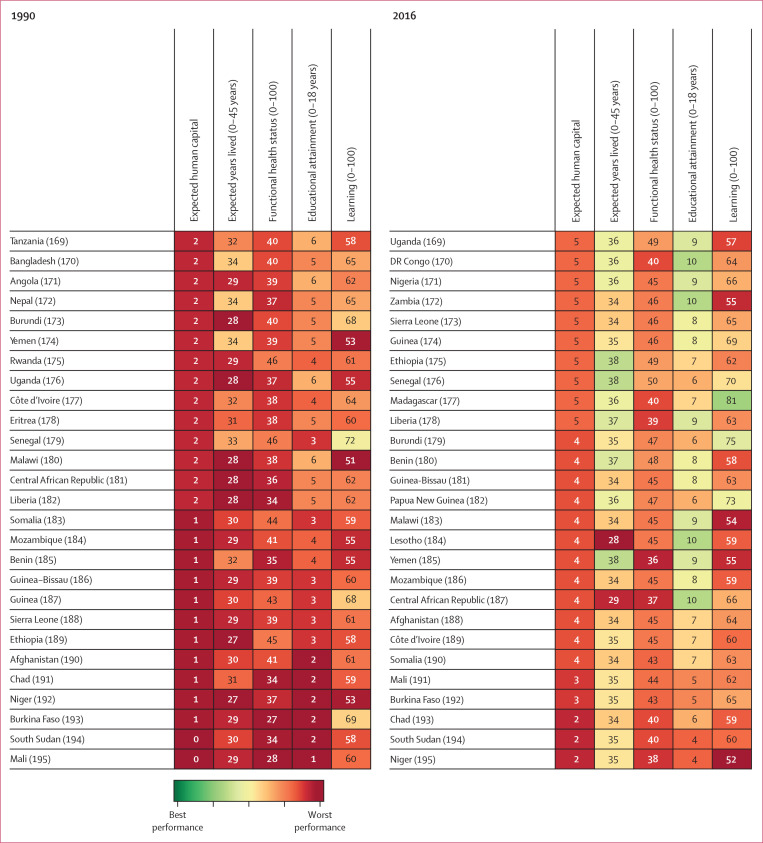
Country rankings and values for expected human capital and for each of its four components, in 1990 and 2016 195 countries are ranked by their expected human capital in 1990 and 2016

**Figure 3:**
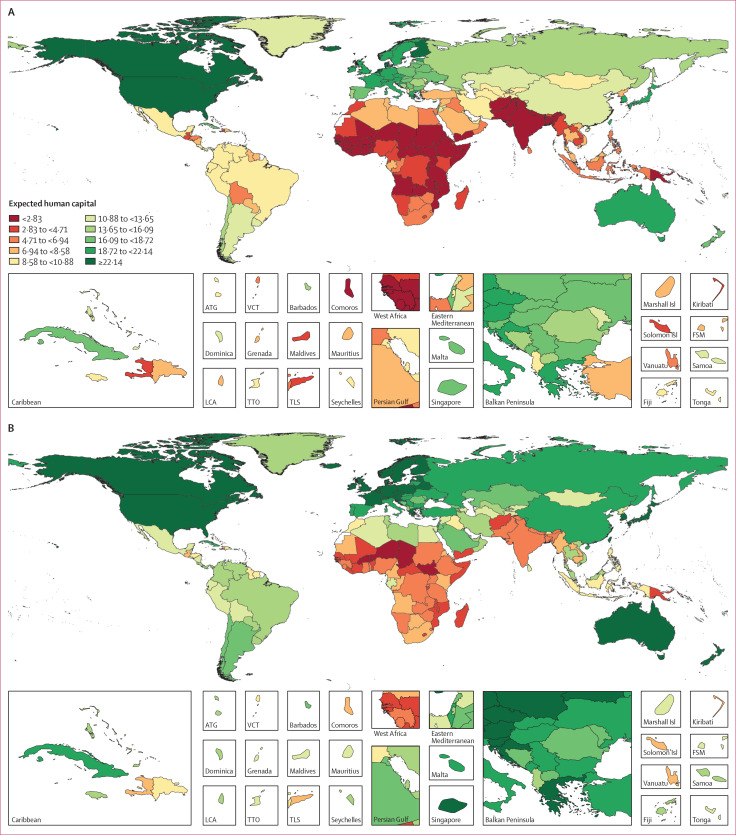
Expected human capital by country in 1990 (A) and 2016 (B) ATG=Antigua and Barbuda. VCT=Saint Vincent and the Grenadines. FSM=Federated States of Micronesia. LCA=Saint Lucia. TTO=Trinidad and Tobago. TLS=Timor-Leste.

In 1990, expected human capital varied widely (figures 2, 3): 16 countries had already achieved more than 20 years of expected human capital, with Finland (24・8 years; 95% UI 24・0–25・6), Iceland (24・1 years; 23・5–24・7), Denmark (23・5 years; 22・9–24・3), Canada (23・1 years; 22・7–23・5), and the Netherlands (22・9 years; 22・4–23・4) being the top five performing countries. Conversely, 61 countries had expected human capital of less than 5 years, including many countries in sub-Saharan Africa and much of south Asia. All countries in Latin America were below 18・5 years of expected human capital. Within western Europe, expected human capital ranged considerably in 1990, from the highest level of 24・8 years (24・0–25・6) in Finland to 14・8 years (14・4–15・3) in Portugal.

Despite 25 years of progress in many dimensions of human capital, in 2016 these levels were not universally high (figures 2, 3). The top five countries were unchanged from 1990 except for the replacement of Canada with Taiwan (province of China). In 2016, all countries in western Europe, and many in central and eastern Europe, had more than 20 years of expected human capital, as did South Korea, Japan, China, Singapore, Taiwan (province of China), Turkey, Brunei, Australia, New Zealand, USA, and Canada. Despite improvements, 24 countries in 2016 continued to have expected human capital below 5 years, with the five lowest-ranked countries being Niger (1・6 years; 95% UI 0・98–2・6), South Sudan (2・0 years; 1・2–3・0), Chad (2・7 years; 1・7–3・2), Burkina Faso (2・8 years; 1・8–4・2), and Mali (2・8 years; 2・0–3・8).

**Figure 4:**
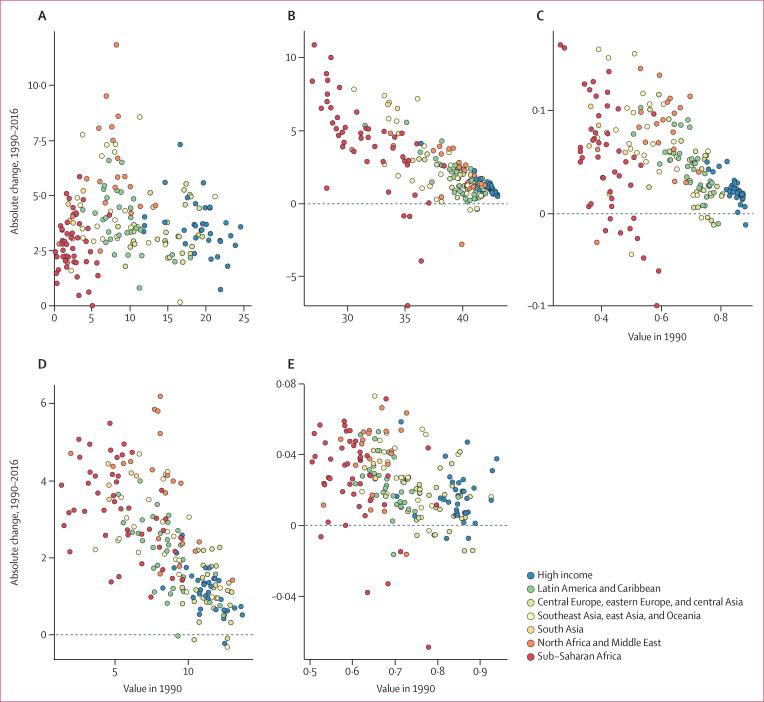
Change in expected human capital and for each of its four components, from 1990 to 2016, compared to 1990 levels Countries categorised into regions according to the Global Burden of Disease super-regions. (A) Expected human capital. (B) Expected years lived, ages 20–64 years. (C) Functional health status. (D) Educational attainment. (E) Learning.

The change in expected human capital between 1990 and 2016 ranged from less than 2 years of progress in 18 countries to more than 5 years of progress in 35 countries (figure 4). For example, the USA, which was ranked sixth in terms of expected human capital in 1990, dropped to rank 27 in 2016 because of minimal progress, particularly on educational attainment. In east and south east Asia, which have generally seen rapid economic growth, many countries had notable improvements South Korea increased from rank 18 in 1990 to rank 6 in 2016; Singapore increased from rank 43 to 13; China increased from rank 69 to 44; Thailand increased from rank 103 to 72; and Vietnam increased from rank 116 to 85.

Differential progress, however, was seen in many other regions. Within Latin America, Brazil had much faster improvements in expected human capital than other countries in the region (improving from rank 91 to 71). The most rapid absolute improvements were seen in several countries in the Middle East (led by Turkey, Saudi Arabia, and Kuwait), although some countries in the region, such as Yemen and Iraq, experienced much slower progress.

Countries had improved expected human capital from 1990 to 2016, and showed changes in each of the four components of expected human capital relative to 1990 levels (figure 4). Although there is a clearer association between improvements in educational attainment and years lived between 20 and 64 years and their respective levels in 1990, these highly differential rates of progress suggest that changes are driven by a combination of policy factors and not just baseline levels. Several countries in north Africa and the Middle East with substantial improvements in expected human capital had a combination of notable increases in educational attainment, learning, and functional health status and to a lesser degree reductions in mortality. A similar picture can be seen for Latin America but at a lower overall magnitude, with improvements driven particularly by increases in educational attainment. In sub-Saharan Africa and to a lesser degree south Asia, improvements in expected human capital are due to improvements in educational attainment and expected years lived in the 20–64 year age range.

**Figure 5:**
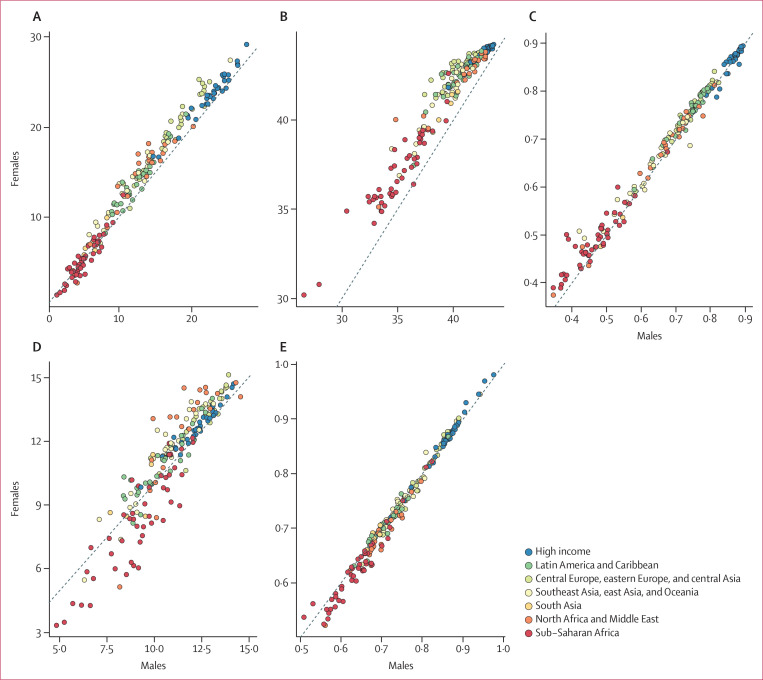
Difference between men and women in expected human capital and for each of its four components, 2016 Countries categorised into regions according to the Global Burden of Disease super-regions. (A) Expected human capital. (B) Expected years lived, ages 20–64 years. (C) Functional health status. (D) Educational attainment. (E) Learning.

Men and women had notable differences in expected human capital in 2016 (figure 5). Across the board, expected years lived between 20 and 64 years were greater in women than men. Similarly, functional health status 2016 was higher among women than men, with the exception of high-income countries. Conversely, learning was higher among men at lower and middle levels of learning—in regions such as sub-Saharan Africa, north Africa and the Middle East, south Asia, and Latin America—but this difference is minimal or non-existent at higher levels and among high-income countries. A clear regional pattern is present for educational attainment, with higher levels for males throughout sub-Saharan Africa and below 9 years of education. In terms of the overall measure of expected human capital, this measure translates into a clear separation at a threshold of 10 years of expected human capital: below this threshold, expected human capital tends to be higher in men whereas above this threshold, expected human capital tends to be higher in women.

### Associations between expected human capital and gross domestic product

**Figure 6:**
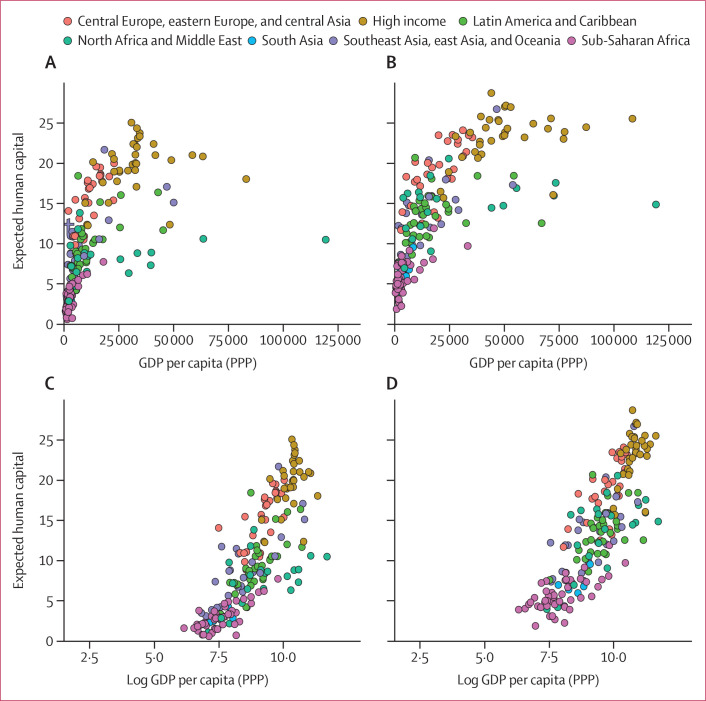
Association between expected human capital and GDP per capita, 1990 and 2016 Countries categorised into regions according to the Global Burden of Disease super-regions. General movement to the right and upwards shows global economic, health, and education development between 1990 and 2016. Expected human capital and GDP per capita in 1990 (A) and 2016 (B). Expected human capital and GDP per capita in log space in 1990 (C) and 2016 (D). GDP per capita is measured in 2017 US$ at PPP. GDP=gross domestic product. PPP=purchasing power parity.

**Figure 7:**
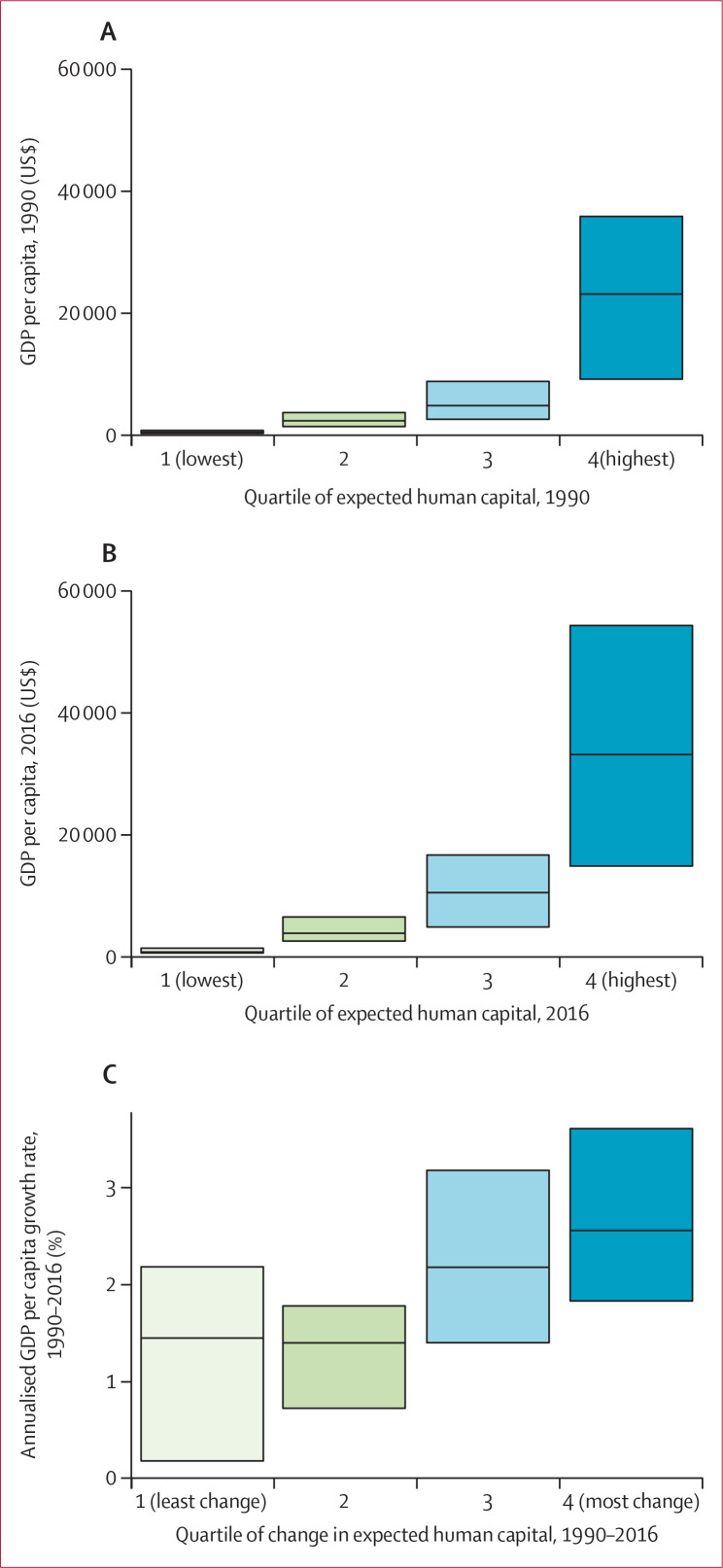
Median and IQR of GDP per capita within each quartile of expected human capital in 1990 (A) and 2016 (B), and median and IQR of annual change in GDP per capita within each quartile of change in expected human capital from 1990 to 2016 (C) GDP=gross domestic product.

We examined the correlation between both levels and change in expected human capital and corresponding levels and change in gross domestic product (GDP) per capita, at the country level (figure 6, 7). Higher levels of expected human capital were associated with higher levels of GDP per capita in both 1990 (figure 6A, 6C, 7A) and 2016 (figure 6B, 6D, 7B). Larger improvements in expected human capital from 1990 to 2016 were also associated with greater GDP growth over the same time period. The top quartile of countries in terms of change in expected human capital from 1990 to 2016 had a median annualised GDP growth of 2・60% (IQR 1・85–3・69) compared with a median annualised GDP growth of 1・45% (0・18–2・19) among the bottom quartile of countries (figure 7C) in terms of change in expected human capital. Although not a formal causal analysis, these differences suggest that both levels of human capital are associated with economic performance and improvements in the production of human capital are associated with faster economic growth.

## Discussion

Our study quantifies levels of human capital in 195 countries from 1990 to 2016, generating a ranking of countries and highlighting huge variations in the production of, and progress in building, human capital across countries. Human capital—educational attainment, learning, functional health, and survival—in 2016 was highest in Finland, Iceland, Denmark, the Netherlands, and Taiwan (province of China), and lowest in Mali, Burkina Faso, Chad, South Sudan, and Niger. Over the past 25 years, progress has been slow in selected countries that started at a high baseline, such as the USA, but perhaps most importantly progress has also been slow in countries with historically low human capital, such as the bottom five countries in 2016. At the macro level, countries that have improved the production of human capital tend to have been more successful in fostering economic growth.

In an article^5^ by World Bank President Jim Y Kim, he states, “with the right measurements, an index ranking the human capital in countries will be hard to ignore, and it can help galvanize much more—and more effective—investments in people”. As part of the Human Capital Project, the World Bank intends to support annual reporting on human capital to keep policy attention focused on investments in health and education that accelerate human capital formation and bring new emphasis to the importance of human capital for economic growth. This study fills this measurement gap by presenting the first ranking of countries by levels of human capital with the use of a comprehensive metric. Although health and education were prominent components of the Millennium Development Goals and remain a focus of the Sustainable Development Goals, the emphasis on human capital signals a shift toward greater consideration of the productive value of health and education, in addition to humanitarian objectives.

By providing an annual measurement of human capital, these rankings can also be used by credit rating agencies in making loan decisions. Agencies that provide independent assessments of risks for national bonds already take into account some measures related to human capital, such as life expectancy.^50^ The markets might incorporate better measures of human capital into borrowing schemes, recognising the challenge of economic growth in settings with low human capital. A virtuous cycle might ensue, in which financial markets reflect future human capital trajectories and create more timely incentives for ministries of finance and other development actors to invest in people today.

Despite wide variations in past rates of human capital accumulation, countries have many available strategies to accelerate progress. To improve average education levels, policy options include reducing or eliminating school fees, shown to increase enrolment and attendance rates in many countries,^51–53^ and carefully targeting infrastructure investments in alignment with needs—eg, building schools in areas with limited access or building latrines, especially for girls. Policies that can improve learning and educational quality include ongoing teacher trainings that incorporate regular follow-up visits and support,^53,54^ improving diagnostics to inform teaching tailored to students’ levels,^55,56^ and grouping students by ability.^57^ To improve survival and the aspects of functional health status studied here, many effective interventions exist for the major infectious diseases: insecticide-treated mosquito nets and artemisinin combination therapy for protection from malaria,^58^ antiretroviral therapy for HIV/AIDS,^59^ directly observed treatment of tuberculosis, ^60^ rotavirus vaccine to prevent diarrhoea,^61^ and pneumococcal vaccine and antibiotics for lower respiratory disease,^62^ among many other cost-effective interventions.^63^ For vision and hearing, effective interventions include vitamin A supplementation to combat childhood blindness,^[Bibr cit0064]64^ corrective lenses, hearing aids, and more advanced technologies, such as cochlear implants. To address chronic malnutrition, available interventions include zinc supplementation and public provision of complementary food for children, and for iron-deficiency anaemia, antenatal micronutrient supplementation and staple food fortification.^65^ Given the wide range of evidence-based policy options with proven effectiveness, the rate of human capital accumulation could accelerate dramatically; however, getting the priority for health and education in national budget discussions correct, might be the main challenge.^27^

Examination of countries with the most rapid improvements in human capital revealed specific policy reforms that probably contributed to observable growth in human capital. Starting in 1995, Brazil implemented a series of education reforms, which included ensuring equal funding across all localities, expanding student testing, and ensuring educational opportunities for poor families, leading to impressive increases in educational attainment. Improving learning is the current national priority, with the aim of achieving Organisation for Economic Co-operation and Development-level test scores by 2021.^66^ The education system in Singapore, which has the highest student test scores in the world, has emphasised quality over the past 20 years, starting with the Thinking Schools, Learning Nation framework in 1997. This encompassed many initiatives to improve learning, including tailoring teaching to students’ level of ability.^67^ Poland’s student performance on international tests dramatically improved after the country implemented educational reforms in 1999, incorporating additional hours of language training and delayed tracking into vocational training.^68^ Thailand was one of the first middle-income countries to achieve universal health coverage, facilitated by the creation of a new public insurance scheme in 2001 and a shift toward greater service provision through primary care centres.^69^ Turkey initiated the Health Transformation Program in 2003, which entailed separating the purchasing and provision of health services, mandating insurance coverage, and reforming provider payment, leading to expanded access and improved patient satisfaction and health outcomes.^70^

Changes in the nature of the world economy, including the increased importance of digital technology, sometimes referred to as the fourth industrial revolution,^[Bibr cit0071]71^ might make human capital even more important for future economic growth. The potentially rising role of highly skilled and healthy workers in the future puts a premium on investing in people now to accelerate human capital formation. This investment requires long-term planning to make major changes in the proportion of children who are healthy and spend up to 18 years attending highquality educational institutions. Health investments can have a double effect on human capital: improved functional health can directly increase the productivity of workers at each age and can also facilitate children attending school and effectively learning. A review^72^ of studies examining the association between early childhood stunting and cognitive development found that an increase of one SD in height-for-age of children younger than 2 years old is associated with an increase of 0・22 SD in cognition at ages 5–11 years. A meta-analysis^73^ of 14 randomised clinical trials of iron supplementation suggested an association between haemoglobin concentration and intelligence, with anaemic premenopausal women experiencing a 2・5-point (95% CI 1・24–3・76) improvement in IQ following iron supplementation.

The estimates of educational attainment presented in this study are highly correlated with other widely used sources, particularly the well known Barro and Lee estimates;^74^ however, the present dataset offers several important advantages over this source and other available sources. First, the data underlying these estimates are based on a systematic synthesis of censuses and household surveys, whereas most other estimates rely heavily on enrolment data, which are subject to a range of inaccuracies; for example, enrolment rates can be well over 100% due to students repeating levels and older learners returning to school. Second, the number of unique data sources informing these estimates is greater than that used in past studies.^75^ Barro and Lee, for example, used 621 unique sources versus the 2522 underlying our estimates.^74^ Third, in the estimation methods, no other study to our knowledge has attempted to parse binned education data—ie, by completed level— into individual years of completed schooling, thereby generating more precise estimates of average years of schooling. Finally, because of the data sources and modelling approach used, we were able to generate annual estimates for 5-year age groups—a level of detail that is not available from most other sources, which more often report estimates in 5-year increments or for more aggregated age groups. One limitation of this analysis to note, however, is that we have not produced estimates of the distribution of educational attainment in countries, only mean levels. The human capital value of educational attainment might not be a linear function of years of education.

The learning estimates presented here would be substantially strengthened through the expansion of international student assessments. Although these results represent the best estimates given all available data, 56 countries have not participated in any internationally similar tests, necessitating substantial reliance on adjusted national tests and covariates to generate a comparable estimate of learning for all countries. Given the importance of educational quality for economic growth, expanding participation in international student tests is a priority for new data collection. Estimates of learning could also be improved with an expansion into quality measures for both tertiary education and on-the-job skills training.

For health, we have used a simple PCA to reduce the complexity of information on several learning-related and productivity-related outcomes, but these PCA weights might not capture the potential for different health outcomes to have differential effects on economic productivity. We have, for example, included some outcomes that are related to cognitive performance, such as stunting, wasting, anaemia, and infectious diseases, but other health outcomes could also be important for productivity, such as mental health and substance abuse. For stunting and wasting, however, we were only able to incorporate period measures of these indicators and not cohort measures because of a paucity of historical data on the prevalence of these conditions. Future work should explore more health outcomes in greater detail and explore their interconnections to economic output.

Formal examination of the association between economic growth and expanded measures of human capital that incorporate broader dimensions, such as the measure presented in this study, is another area of future work. In this study, we examined associations between levels and trends in our measure of human capital and levels and trends in GDP. Although these simple analyses suggest a correlation, we do not make claims of causality because they are not causal analyses. Future work in this area will need to address the potential problem of reverse causality. In other words, do improvements in human capital lead to faster economic growth or does faster economic growth allow countries to better invest in human capital?

Our study focused on national levels of human capital, but geospatial analyses have shown disparities in average years of schooling.^76^ In future work, we believe it will be a useful planning tool to measure human capital at this high spatial resolution. Within a geographical location, measurement of mean levels of human capital might not be enough; measurement of the full distribution will allow testing of hypotheses about the comparative importance of secondary and tertiary education access and quality. Such granular information could be used to target communities that are the worst off and to assess efforts to reduce inequalities as part of the Sustainable Development Goals framework.

The World Bank argues that countries are not investing enough in health and education to benefit from the potential of their own human capital. We provide the first comprehensive assessment of expected human capital for 195 countries from 1990 to 2016. Countries have varied substantially in the pace of improving human capital, holding out the promise that wider implementation of targeted policies and funding focused on improving health and education can accelerate human and economic development.

### Contributors

SSL and CJLM managed the estimation process. CJLM, KC, SSL, EG, RLU, and HY wrote the first draft of the manuscript. CJLM, SSL, RLU, ASK, HY, RMB, and JD developed the methods or computational machinery. RLU, ASK, HY, RMB, and JF applied analytical methods to produce estimates. ATL, YR, HJT, JLW, RX, RLU, and KC extracted, cleaned, or catalogued data, and designed or coded figures and tables. MFB, SSL, CJLM, and EG managed the overall process.

### Declaration of interests

We declare no competing interests.
